# Inositol possesses antifibrotic activity and mitigates pulmonary fibrosis

**DOI:** 10.1186/s12931-023-02421-6

**Published:** 2023-05-16

**Authors:** Ji-Min Li, Wen-Hsin Chang, Linhui Li, David C. Yang, Ssu-Wei Hsu, Nicholas J. Kenyon, Ching-Hsien Chen

**Affiliations:** 1 Division of Pulmonary, Critical Care, and Sleep Medicine, Department of Internal Medicine, University of California Davis, Davis, CA USA; 2 Division of Nephrology, Department of Internal Medicine, University of California Davis, Davis, CA 95616 USA

**Keywords:** IPF, Lung fibroblasts, Fibrometabolism, Myo-inositol, ASS1

## Abstract

**Background:**

Myo-inositol (or inositol) and its derivatives not only function as important metabolites for multiple cellular processes but also act as co-factors and second messengers in signaling pathways. Although inositol supplementation has been widely studied in various clinical trials, little is known about its effect on idiopathic pulmonary fibrosis (IPF). Recent studies have demonstrated that IPF lung fibroblasts display arginine dependency due to loss of argininosuccinate synthase 1 (ASS1). However, the metabolic mechanisms underlying ASS1 deficiency and its functional consequence in fibrogenic processes are yet to be elucidated.

**Methods:**

Metabolites extracted from primary lung fibroblasts with different ASS1 status were subjected to untargeted metabolomics analysis. An association of ASS1 deficiency with inositol and its signaling in lung fibroblasts was assessed using molecular biology assays. The therapeutic potential of inositol supplementation in fibroblast phenotypes and lung fibrosis was evaluated in cell-based studies and a bleomycin animal model, respectively.

**Results:**

Our metabolomics studies showed that ASS1-deficient lung fibroblasts derived from IPF patients had significantly altered inositol phosphate metabolism. We observed that decreased inositol-4-monophosphate abundance and increased inositol abundance were associated with ASS1 expression in fibroblasts. Furthermore, genetic knockdown of ASS1 expression in primary normal lung fibroblasts led to the activation of inositol-mediated signalosomes, including EGFR and PKC signaling. Treatment with inositol significantly downregulated ASS1 deficiency-mediated signaling pathways and reduced cell invasiveness in IPF lung fibroblasts. Notably, inositol supplementation also mitigated bleomycin-induced fibrotic lesions and collagen deposition in mice.

**Conclusion:**

These findings taken together demonstrate a novel function of inositol in fibrometabolism and pulmonary fibrosis. Our study provides new evidence for the antifibrotic activity of this metabolite and suggests that inositol supplementation may be a promising therapeutic strategy for IPF.

**Supplementary Information:**

The online version contains supplementary material available at 10.1186/s12931-023-02421-6.

## Background

Idiopathic pulmonary fibrosis (IPF) is the most frequent interstitial lung disease with chronic and progressive features that lead to decline in lung function and ultimately lung failure [1, 2]. The fibrotic lesions observed in IPF are thought to be primarily driven by fibroblasts, particularly myofibroblasts, which are the major cell types responsible for fibrotic progression. This process is mediated through formation of focal lesions of active fibroblasts, exuberant production and deposition of extracellular matrix (ECM), which ultimately leads to thickening of the interstitium and distortion of the lung architecture [3–5]. The disease exhibits a median survival of less than 5 years from the time of diagnosis due to a lack of effective therapies [6, 7]. While two currently available treatment options, nintedanib and pirfenidone, can improve the lung function in patients, both drugs fail to effectively halt lung fibrosis, with little to no effect on overall mortality [7, 8]. For these reasons, there is an urgent need to develop new and better therapeutic options for those diagnosed with IPF.

Recent studies demonstrate that dysregulated expression of metabolic enzymes and altered levels of metabolites are involved in IPF pathogenesis [9–11] where metabolic reprogramming modulates the fibrotic activities of lung fibroblasts [12]. Inhibition of glycolysis, glutaminolysis or arginine biosynthesis has been shown to reduce pulmonary fibrosis in animal models, suggesting targeting metabolic reprogramming of fibrotic lung fibroblasts as a viable therapeutic avenue for IPF [13–16]. We have previously identified downregulation of argininosuccinate synthase 1 (ASS1), a rate-limiting enzyme in *de novo* arginine biosynthesis, in IPF lung fibrotic tissues and fibroblasts. As IPF lung fibroblasts with ASS1 deficiency require the uptake of extracellular arginine for survival due to an inability to endogenously synthesize arginine, arginine deprivation strategies, including arginine-free diet and arginine deiminase (ADI) which degrades extracellular arginine, demonstrate antifibrotic effects on reducing collagen content and fibrotic lesions in the lung of mice exposed to bleomycin [16]. Although ADI therapy exhibited promising results, several studies in cancer have reported that this therapy inevitably triggers ADI resistance through induction of ASS1 expression and activation of alternate signaling pathways in ASS1-negative cells [17, 18]. Given these shortcomings, the development of novel therapeutic strategies for arginine-dependent pulmonary fibrosis is greatly needed.

Myo-inositol (or inositol) is a natural compound provided by dietary uptake and/or endogenously synthesized through the glycolysis pathway [19]. As a precursor of second messengers, inositol regulates an array of signal transduction pathways and metabolic circuits in cells [20, 21]. One of the most common inositol-derived metabolites, phosphatidylinositol 4,5-bisphosphate (PIP_2_), hydrolyzes into diacylglycerol (DAG) and inositol 1,4,5-trisphosphate (IP3) to mediate Ca^2+^ signaling and activation of the conventional protein kinase C isoforms (cPKCs) [21, 22]. Since inositol metabolism plays a critical role in different aspects of physiological conditions, inositol has been considered for treating a diverse range of conditions from newborn respiratory distress syndrome to lung cancer [23, 24]. Despite these findings, our understanding of inositol function in other lung diseases such as lung fibrosis remains incomplete. In this study, we not only characterized the metabolomic profiles of fibrotic lung fibroblasts derived from IPF patients but also identified fibrogenic metabolic pathways driven by ASS1 deficiency. Furthermore, we determined the therapeutic potential of inositol in IPF through in-vitro cell-based assays and an in-vivo animal model of lung fibrosis.

## Materials and methods

### Reagents and antibodies

All reagents and antibodies used in this study are described in the Supplementary Methods in the online supplement.

### Cell culture and transfection

Human primary fibroblast cells were obtained from airway tissues provided from the UC Davis Medical Center (Sacramento, CA) with consent as previously described [16]. The protocol for human tissue procurement and usage were periodically reviewed and approved by the UC Davis Institutional Review Board. Detailed experimental procedures for establishment of cell culture and siRNA transfections are described in the Supplementary Methods in the online supplement.

### Untargeted GC-TOF MS analysis

Frozen lung fibroblast samples were submitted to West Coast Metabolomics Center at UC Davis, and the cells were analyzed using gas chromatography/time-of-flight mass spectrometry (GC-TOFMS) analysis as previously described [25]. Detailed experimental procedure for GC-TOF MS and data analysis are described in the Supplementary Methods in the online supplement.

### Myo-inositol assays

The content of inositol in lung fibroblast cells was measured by using a myo-inositol assay kit according to the manufacturer’s protocol (Abcam, Cambridge, MA). Detailed experimental procedure is described in the Supplementary Methods in the online supplement.

### Bleomycin-induced pulmonary fibrosis model

8-week-old C57BL/6J female mice purchased from The Jackson Laboratory (Bar Harbor, ME, USA) were housed 4 mice per cage and fed with rodent laboratory chow *ad libitum*. Saline or bleomycin was intratracheally administered to C57BL/6J mice as previously described [16, 26, 27]. Detailed experimental procedures are described in the Supplementary Methods in the online supplement. The procedures of all mouse experiments were approved by the Institutional Animal Care and Use Committee (IACUC) of UC Davis.

### Immunoblotting, quantitative real-time polymerase chain reaction (PCR), hydroxyproline analysis, and cell-based assays

Detailed procedures for immunoblotting, quantitative real-time polymerase chain reaction (PCR), hydroxyproline analysis, and cell-based assays including cell viability, colony formation, and cell invasion analyses are described in the Supplementary Methods in the online supplement. The primers used in quantitative real-time PCR analysis were listed in Additional file 1: Table S1.

### Statistical analysis

Data are represented as the mean ± standard error (SE) from at least three independent experiments. Statistical differences of data in vitro and in vivo were assessed using an unpaired two-tailed Student’s t test and a one-way analysis of variance (ANOVA) test. In survival analysis, overall survival curves for groups were obtained by the Kaplan–Meier method and the differences in survival among groups were analyzed using the log-rank test. All analyses were performed using SPSS software (v.10.0; SPSS, Chicago, IL, USA). For the metabolomics analysis, the significance of differences in the mean value was assessed by two-sample Hotelling’s *T*^*2*^ statistics built in MetaboAnalyst 5.0. All test results with *p* values < 0.05 were considered statistically significant.

## Results

### Metabolic changes in fibrotic lung fibroblasts

**Fig. 1:**
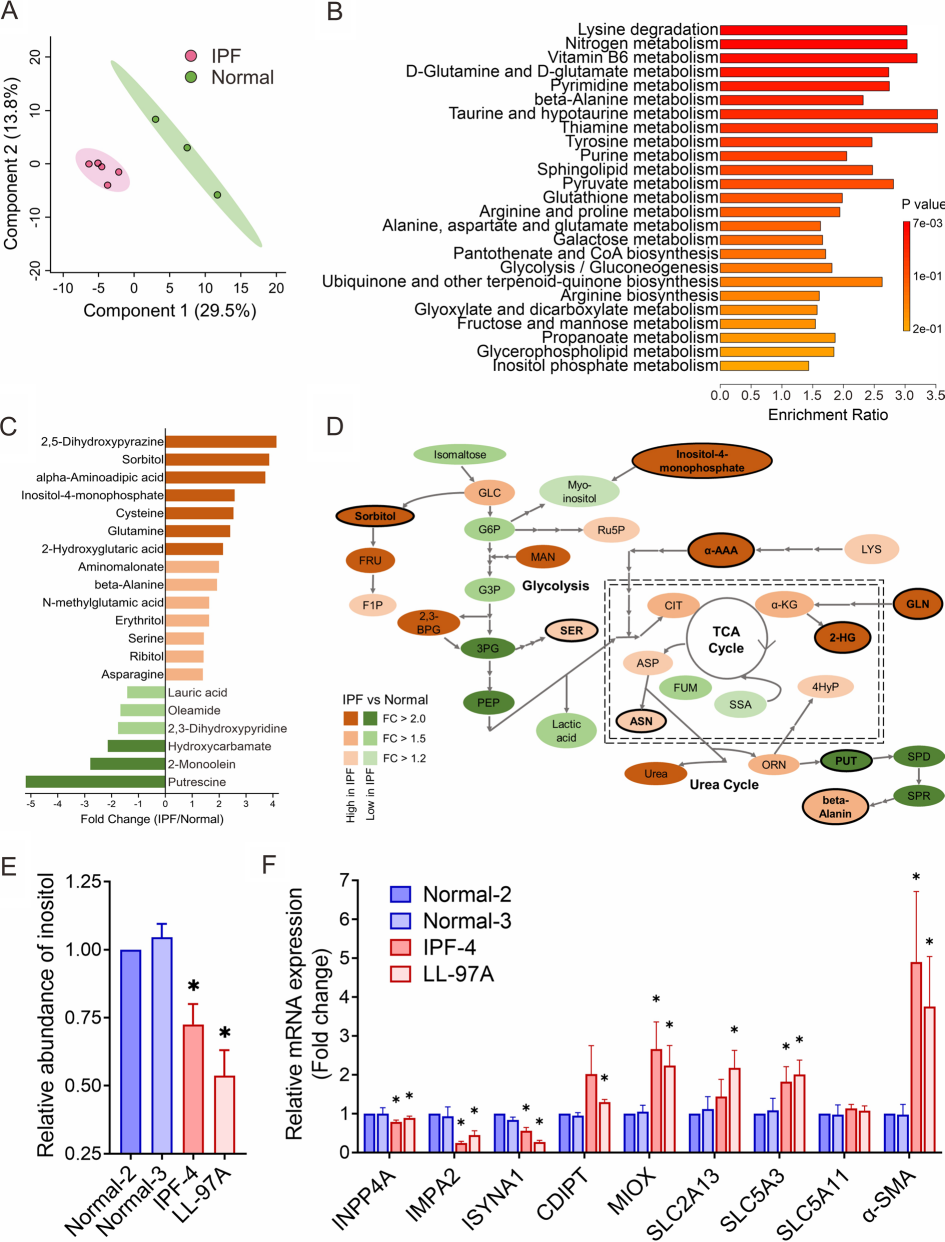
Differential metabolic pathways and metabolites between normal and IPF lung fibroblasts. **A** Partial least-squares discriminant analysis (PLS-DA) of the metabolite values across normal (green) and IPF (pink) primary lung fibroblasts. **B** Top 25 ranking enriched differential metabolic pathways between normal and IPF primary lung fibroblasts identified by Metabolite Set Enrichment Analysis (MSEA). **C** Significantly differential metabolites between normal and IPF primary lung fibroblasts. Orange: higher abundance in IPF fibroblasts. Green: higher abundance in normal fibroblasts. **D** Metabolic alterations of glycolysis, TCA cycle and urea cycle between normal and IPF primary lung fibroblasts. Color-coded metabolites correspond to the fold changes between normal and IPF lung fibroblasts. *GLC* glucose, *G6P* glucose 6-phosphate, *G3P* glyceraldehyde 3-phosphate, *3PG* 3-phosphoglycerate, *2,3-BPG* 2,3-Bisphosphoglyceric acid, *PEP* phosphoenolpyruvate, *PYR* pyruvate, *FRU* fructose, *F1P* fructose 1-phosphate, *Ru5P* ribulose-5-phosphate, *MAN* Mannose, *SER* serine, *LYS* lysine, *α-AAA* α-aminoadipic acid, *CIT* citrate, *α-KG* α-ketoglutarate, *GLN* glutamine, *2-HG* 2-hydroxyglutarate, *SSA* succinic semialdehyde, *FUM* fumarate, *ASP* aspartate, *ASN* asparagine, *ORN* ornithine, *4HyP* 4-hydroxyproline; *PUT* putrescine, *SPD* spermidine, *SPR* spermine. **E** The abundance of inositol in normal and IPF lung fibroblasts was determined by using inositol assays (mean ± SE, n = 3, *p < 0.05 versus Normal-2). **F** mRNA expression levels of the enzymes involved in inositol biosynthesis (INPP4A, IMPA2, and ISYNA1), inositol catabolism and phosphatidylinositol metabolism (MIOX and CDIPT), and inositol transporters (SLC2A13, SLC5A3, and SLC5A11) were measured by RT-qPCR analysis (mean ± SE, n = 3; *p < 0.05 versus Normal-2)

Given the emerging role of metabolic reprogramming in IPF pathogenesis, we aimed to reveal potential IPF-specific metabolic pathways and/or novel elements of fibrogenic pathways. We subjected primary lung fibroblasts derived from IPF patients (IPF, n = 5) and postmortem lung tissues from healthy individuals (normal, n = 3) to untargeted metabolomics and identified 159 metabolites in both groups. Metabolites analyzed by the partial least squares discriminant analysis (PLS-DA) algorithm resulted in visually separated clusters between IPF and normal groups (Fig. 1A), suggesting that the metabolite profiles were significantly different between IPF and normal lung fibroblasts. Metabolite set enrichment analysis (MSEA) identified the top 25-metabolic pathways significantly altered in IPF (Fig. 1B). Among these top 25 pathways, 9 metabolic pathways have been well-documented in previous metabolomics studies in IPF, including glutamine metabolism, arginine metabolism and sphingolipid metabolism [11]. Additionally, 8 out of the 25 pathways were amino acid-associated metabolic pathways, of which the beta-alanine, taurine and hypotaurine, and inositol phosphate metabolism pathways have been reported to participate in the pathogenesis of lung diseases [28–30]. Despite these findings, the roles these metabolites play in lung fibrosis remain enigmatic.

Of the significant metabolites between normal and IPF cells, we noticed that putrescine was the most abundant metabolite in normal fibroblasts, whereas sorbitol was highly abundant in IPF (Fig. 1C). alpha-Aminoadipic acid (α-AAA), a metabolite of lysine degradation, was also elevated in the IPF group, suggesting a potential metabolic flux from lysine metabolism into the tricarboxylic acid (TCA) cycle. Additionally, inositol-4-monophosphate, cysteine and glutamine had a fold change greater than 2.4 in IPF relative to normal fibroblasts. Since most of the significant metabolites are involved in the pathways branched from glycolysis or TCA cycle, we constructed a metabolic map with fold changes for each significant metabolite (Fig. 1D). The metabolic network showing a general downregulation of glycolysis was noted in IPF lung fibroblast cells. The intermediates of glycolytic breakdown could be directed to the sorbitol, pentose phosphate pathway and myo-inositol (or inositol) metabolism.

In contrast to the overall upregulated sorbitol pathway, the metabolite inositol was decreased, followed by an upregulated inositol-4-monophosphate (Fig. 1D). This shift in metabolite abundance levels from inositol to inositol-4-monophosphate suggests dysregulation of inositol metabolism in IPF fibroblasts. We next performed inositol assays to confirm the abundance of cellular inositol in lung fibroblasts. Figure 1E shows that cellular inositol abundance was significantly reduced in IPF lung fibroblasts (IPF-4 and LL-97A) as compared to normal fibroblasts. Moreover, treatment with TGF-β, a major profibrogenic factor, significantly decreased cellular inositol abundance in normal fibroblasts, Normal-2 and Normal-3 (Figure S1A). These results taken together provide evidence of inositol downregulation in fibrotic lung fibroblasts.

### Loss of ASS1 expression rewires inositol-associated metabolic pathways

Given that inositol could be synthesized through biochemical pathways in cells and/or be imported into the cells by transporters, we first assessed the expression levels of multiple enzymes involved in inositol biosynthesis or catabolism in lung fibroblasts. Using quantitative real-time PCR (RT-qPCR) analysis, we demonstrated an obvious decrease in mRNA expression levels of the enzymes associated with inositol biosynthesis, including inositol polyphosphate-4-phosphatase type I A (INPP4A), inositol monophosphatase 2 (IMPA2), and inositol-3-phosphate synthase 1 (ISYNA1) as well as a significant increase in the gene expression of myo-inositol oxygenase (MIOX) which is involved in inositol catabolism in IPF fibroblasts compared to normal fibroblasts (Fig. 1F). Elevated expression of CDP-diacylglycerol-inositol 3-phosphatidyltransferase (CDIPT), an enzyme associated with phosphatidylinositol metabolism, was also seen in IPF fibroblasts. As expected, both MIOX and CDIPT mRNA levels were upregulated in normal fibroblasts upon TGF-β treatment (Additional file 1: Fig. S1B). These findings indicate the possibility that lower inositol abundance may be attributed to dysregulated expression of the enzymes associated with inositol biosynthesis and catabolism.

**Fig. 2:**
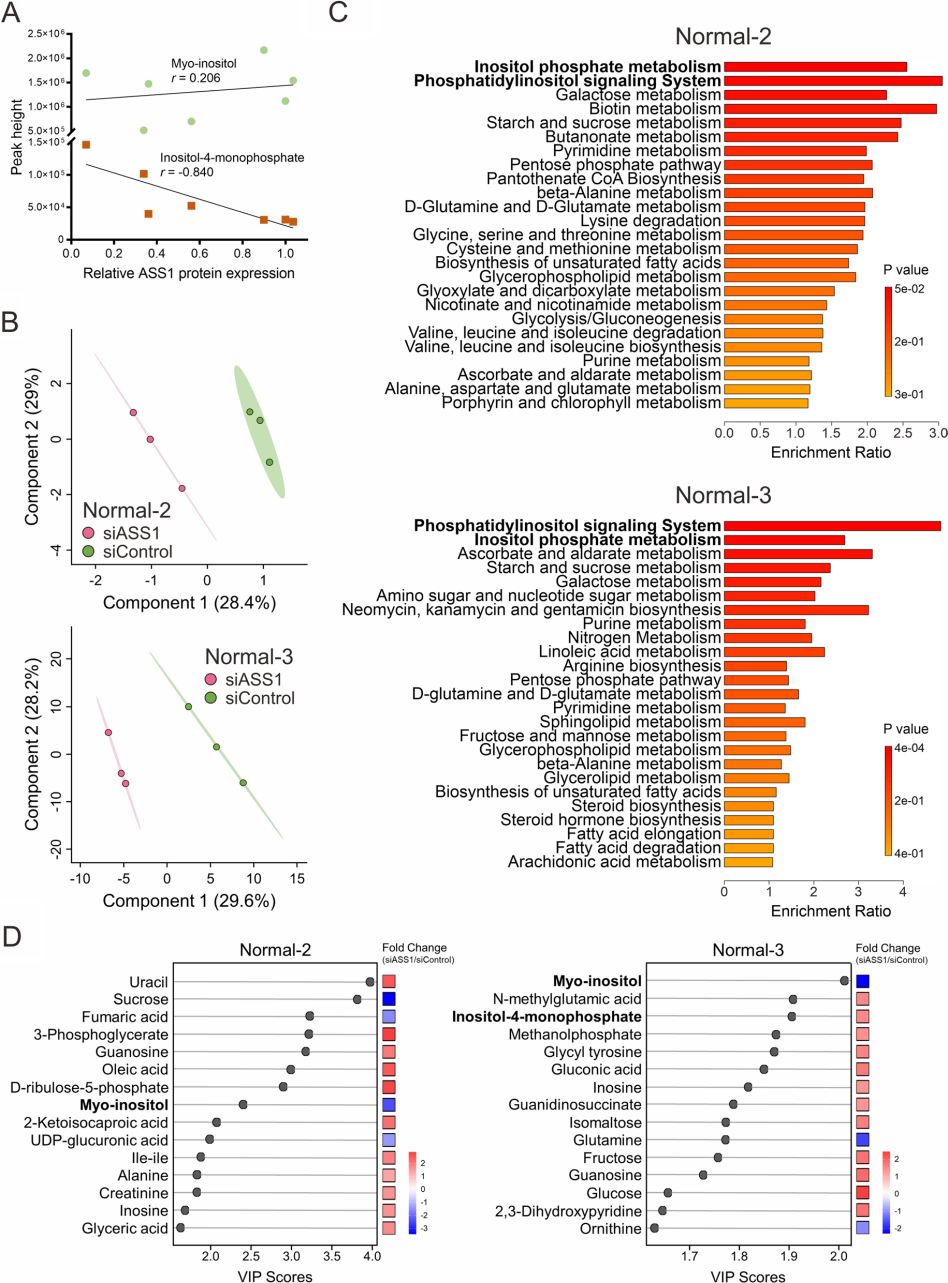
ASS1-mediated metabolic changes in primary lung fibroblasts. **A** The correlation of ASS1 protein expression and myo-inositol (or inositol) abundance (green dot) or ASS1 protein expression and inositol-4-monophospahte abundance (orange square) from three normal fibroblasts and four IPF fibroblasts. The solid black lines represent the linear relationship. *r* is the Pearson correlation coefficient. **B** PLS-DA of the metabolite values across the normal primary lung fibroblasts transfected with either non-targeting control siRNAs (siControl) or ASS1-specific siRNAs (siASS1). **C** Top 25 ranking enriched differential metabolic pathways between control and ASS1-knockdown lung fibroblasts identified by Metabolite Set Enrichment Analysis (MSEA). **D** Variable importance in the projection (VIP) score plots for the top-15 most important metabolites identified by PLS-DA in (**B**). The boxes on the right side indicate the relative abundance in each group. Red: higher abundance; Blue: lower abundance

Given our findings in inositol biosynthesis and catabolism, we next turned our attention to the cellular transport of inositol. Through the analysis of the transcriptome dataset GSE124685, we found several inositol transporter genes are dysregulated in IPF lung tissues. Compared to normal lung tissues, expression of a Na^+^-coupled inositol transporter, SMIT1 (SLC5A3), was ubiquitously upregulated in all IPF lung tissues from all stages of IPF, while only a slight decrease of the proton coupled inositol transporter HMIT (SLC2A13) was seen in lung tissues from end-stage IPF (Additional file 1: Fig. S2A). In contrast to the findings in whole lung tissues, our RT-qPCR data confirmed elevated mRNA expression of SLC5A3 and SLC2A13 in IPF lung fibroblast cells, despite no difference in SMIT2 (SLC5A11) mRNA levels between IPF and normal fibroblasts (Fig. 1F). Similarly, TGF-β treatment upregulated mRNA expression of a number of inositol transporters such as SLC5A3 and SLC5A11 (Additional file 1: Fig. S1B), supporting the concept of dysregulated inositol transport and metabolism in IPF pathogenesis. In light of the prior observations [15, 16] that a majority of IPF lung fibroblasts are deficient in ASS1 (Additional file 1: Fig. S3A), we examined the interaction between inositol metabolism and ASS1 expression. Inositol-4-monophosphate, a major inositol precursor, displayed a significant inverse correlation with ASS1 expression level, whereas inositol abundance was positively associated with ASS1 expression (Fig. 2A).

With our observations that inositol is correlated with ASS1, we set to characterize the metabolic pathways regulated by ASS1 and determine that ASS1 expression indeed modulates inositol metabolism. To accomplish this, we utilized an ASS1-specific small interfering RNA (siRNA) to deplete endogenous ASS1 in primary normal lung fibroblasts (Normal-2 and Normal-3) and conducted untargeted metabolomic studies of primary normal lung fibroblasts with and without ASS1 knockdown (n = 3). The PLS-DA analysis demonstrated altered metabolite levels and segregation of control (siControl) and ASS1-knockdown (siASS1) groups (Fig. 2B). Metabolite set enrichment analysis (MSEA) confirmed that two of inositol-associated metabolic pathways, inositol phosphate metabolism and phosphatidylinositol signaling system, were the highest-ranked metabolic pathways in ASS1-deficient lung fibroblasts (Fig. 2C). We next assessed the significant metabolites and found that inositol was remarkably downregulated in ASS1-knockdown cells, suggesting the crucial role of inositol metabolism in lung fibroblasts with ASS1 loss (Fig. 2D and Additional file 1: Fig. S4A). These data suggest that ASS1 loss likely reprograms inositol-related metabolic pathways in lung fibroblasts.

### ASS1 deficiency activates inositol-associated signaling molecules

**Fig. 3:**
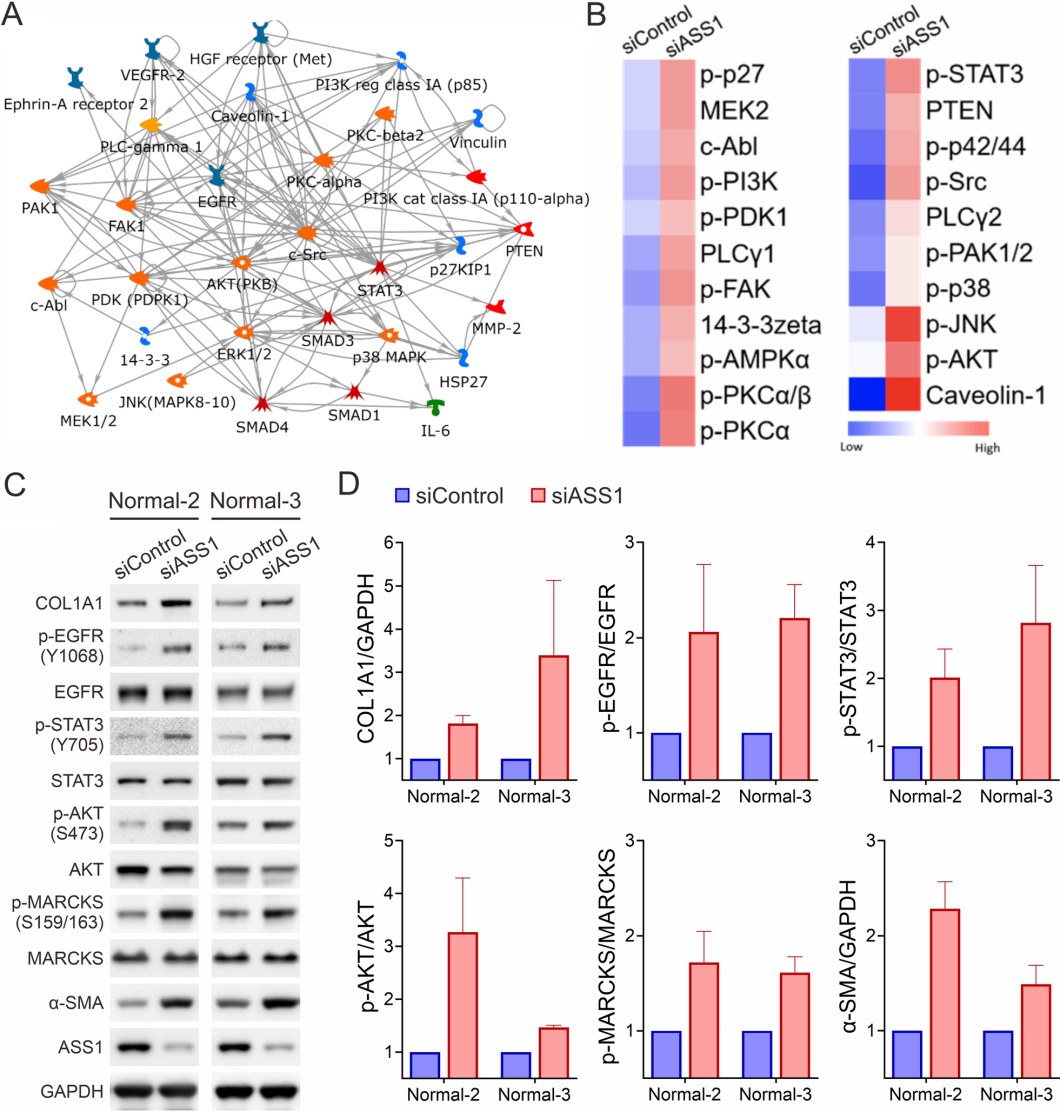
Upregulation of signaling cascades and profibrotic markers in ASS1-deficient normal lung fibroblasts. **A** A protein network of inositol-responsive signaling pathways in ASS1-deficient lung fibroblasts identified by the MetaCore™ analytical suite. **B** A heatmap graph of the differentially expressed proteins involved in the inositol signaling pathway from the reverse-phase protein array (RPPA) profiles in control and ASS1-knockdown normal lung fibroblasts. **C** Immunoblotting analysis of inositol-responsive molecules and profibrotic markers in control and ASS1-knockdown normal lung fibroblasts. **D** Normalized expression levels of inositol-responsive molecules and profibrotic markers from (**C**). Data are represented as mean ± SE, n = 3

Given the alterations observed in inositol metabolism and the relationship of this metabolic pathway with cellular signaling, we profiled signaling networks regulated by ASS1 in complement to our metabolomic studies. Both control and ASS1-knockdown cells were subjected to reverse phase protein arrays (RPPAs), followed by analysis with the Metacore networks. Consistently, these network analyses revealed multiple interactions among inositol-associated signaling molecules that were significantly affected by ASS1 (Fig. 3A). 31 molecules were shown to form interaction loops among different molecules, the majority of which were downstream of the epidermal growth factor receptor (EGFR) and PKC pathways where inositol-derived second messengers PIP2, IP3, and DAG, are required to fully activate the pathways [31, 32]. Inositol-associated kinases, which function in inositol phosphate metabolism and are mainly involved in the inositol trisphosphate signaling, were activated in ASS1-knockdown cells (Fig. 3B). Of particular note, ASS1-deficient lung fibroblasts exhibited higher phosphorylation levels of PLC, cPKCs, and PI3K/AKT. We next evaluated the activity of the most common inositol-associated signaling molecules regulated by ASS1. Immunoblotting analysis confirmed higher phosphorylation levels of EGFR, AKT, STAT3, and MARCKS, a major substrate of cPKCs, as well as elevated expression of fibrotic molecules including collagen type I alpha 1 (COL1A1) and alpha smooth muscle actin (α-SMA) in lung fibroblasts upon ASS1 knockdown (Fig. 3C, D). These observations support the notion of the role of ASS1 in inositol-associated signaling activity.

### Inositol treatment inhibits inositol-associated signaling activity and cell invasiveness

**Fig. 4:**
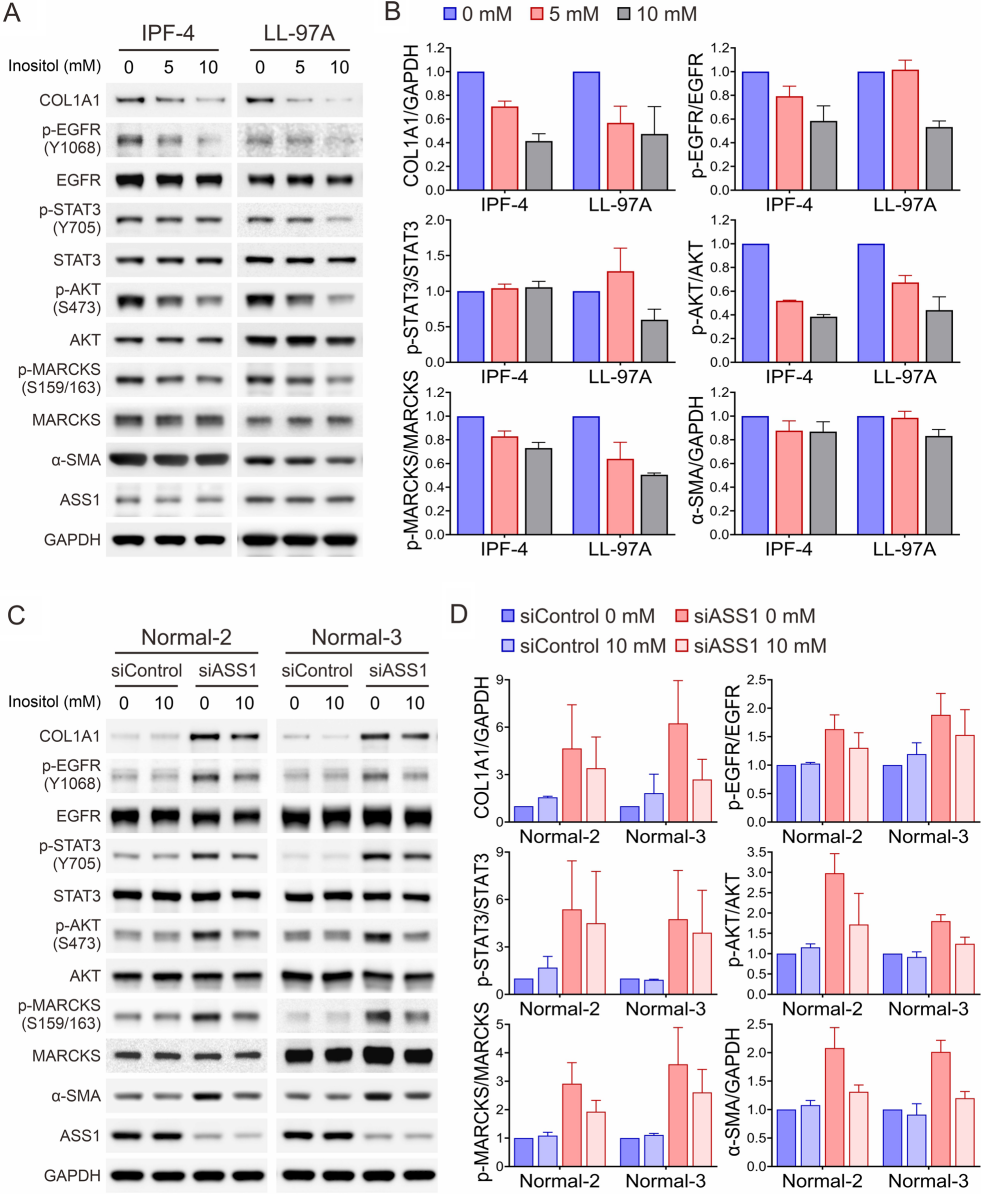
Responsiveness to inositol exposure in IPF and ASS1-deficient normal lung fibroblasts. **A** Representative Western blot images of inositol-responsive molecules and profibrotic markers in IPF lung fibroblasts upon exposure to inositol. **B** Quantification of protein expression levels from (**A**) using ImageJ software. Data are expressed as mean ± SE, n = 3. **C** Representative Western blot images of inositol-responsive molecules and profibrotic markers in control and ASS1-knockdown normal fibroblasts treated with or without inositol. **D** Quantification of protein expression levels from (**C**) using ImageJ software. Data are expressed as mean ± SE, n = 3

Since abundance of inositol was decreased and inositol-related signaling molecules were activated in IPF and/or ASS1-deficient lung fibroblast cells, we asked whether inositol supplementation could alter cellular metabolism and thereby disrupt inositol-driven signaling. To this end, two IPF lung fibroblasts with low ASS1 expression, IPF-4 and LL-97A cells [16], were incubated with various doses of inositol (0, 5, and 10 mM) for 24 h and subjected to immunoblotting analysis. Treatment with inositol suppressed phosphorylation levels of inositol-related signaling molecules (e.g., EGFR, AKT, STAT3, and MARCKS) and expression of COL1A1 (Fig. 4A). Treatment also suppressed AKT phosphorylation, a common downstream molecule between EGFR and PKC signaling. Suppression of these molecules appeared to be concentration-dependent (Fig. 4B). Further confirmation of this phenomenon was observed in ASS1-knockdown normal fibroblasts, with several molecules such as COL1A1, α-SMA, and AKT phosphorylation induced by ASS1 knockdown being repressed in response to inositol treatment (Fig. 4C, D).

**Fig. 5:**
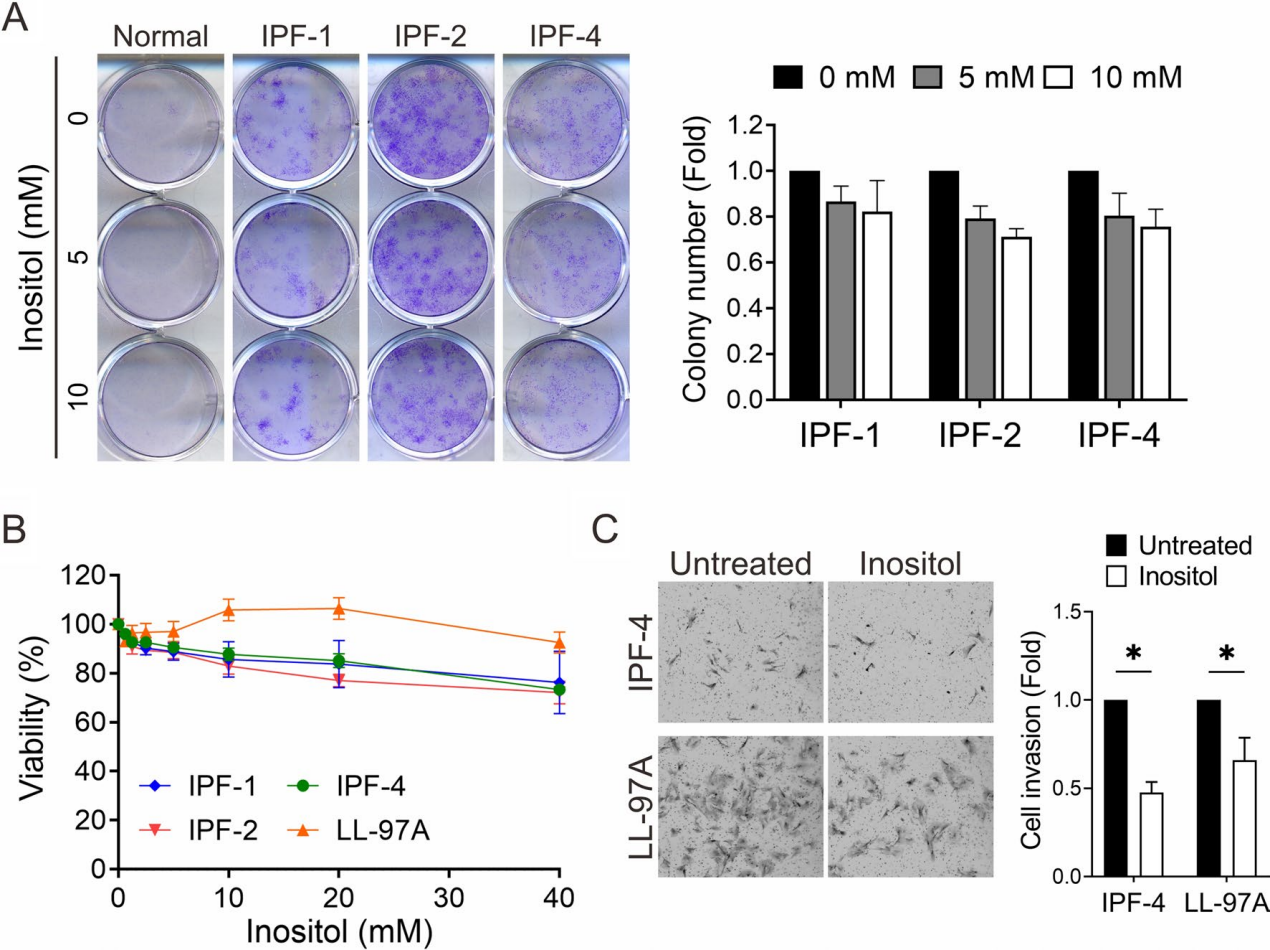
Functionality of inositol in IPF lung fibroblasts. **A** Effect of inositol exposure on the colony-forming ability of normal and IPF primary lung fibroblasts. Cells were seeded to grow and treated with indicated concentration of inositol for 14 days. Left: representative images of cell colonies. Right: quantification of colonies stained by crystal violet and counted under phase microscopy (mean ± SE, n = 3). **B** Cell viability of IPF lung fibroblasts treated with inositol. IPF lung fibroblasts were incubated in various concentrations of inositol for 72 h, and cell viability was assessed by a CCK8 assay (mean ± SE, n = 3). **C** Analysis of cell invasion ability of inositol-treated IPF lung fibroblasts determined by Matrigel invasion assays. Left: representative images of stained invaded cells. Right: quantification of invaded cell number (mean ± SE, n = 3, *p < 0.05 versus untreated group)

The majority of lung fibroblasts from IPF patients display an abnormally activated (pro-fibrotic) phenotype, namely pathological alterations in proliferation, invasion, and differentiation into myofibroblasts [33–35]; therefore, we next examined whether inositol inhibits fibroblast cell proliferation and invasion, two important determinants of fibrosis progression. One normal lung fibroblast cell (Normal) and three IPF lung fibroblast cells (IPF-1, -2, and − 4) were subjected to colony-forming assays after incubation with various doses of inositol (0, 5, and 10 mM). Analysis showed no significant inhibitory effect on cell proliferation and clonogenic ability in cells treated with inositol (Fig. 5A). Additionally, four IPF lung fibroblasts, IPF-1, -2, -4, and LL-97A cells, were exposed to increasing concentrations of inositol from 0 to 40 mM and subjected to cell counting kit 8 (CCK8) cell viability assays after 72 h. Inositol treatment demonstrated a modest effect on cell viability in three of the tested IPF lung fibroblasts (Fig. 5B). Next, both IPF-4 and LL-97A cells were selected given their high invasive ability as described previously [16], and treated with inositol for 48 h. Matrigel transwell invasion assays showed suppression in cell invasiveness in the primary IPF fibroblasts in response to inositol (Fig. 5C). These data support the anti-fibrogenic properties of inositol in fibrotic lung fibroblasts.

### Inositol administration attenuates bleomycin-induced lung fibrosis in mice

**Fig. 6:**
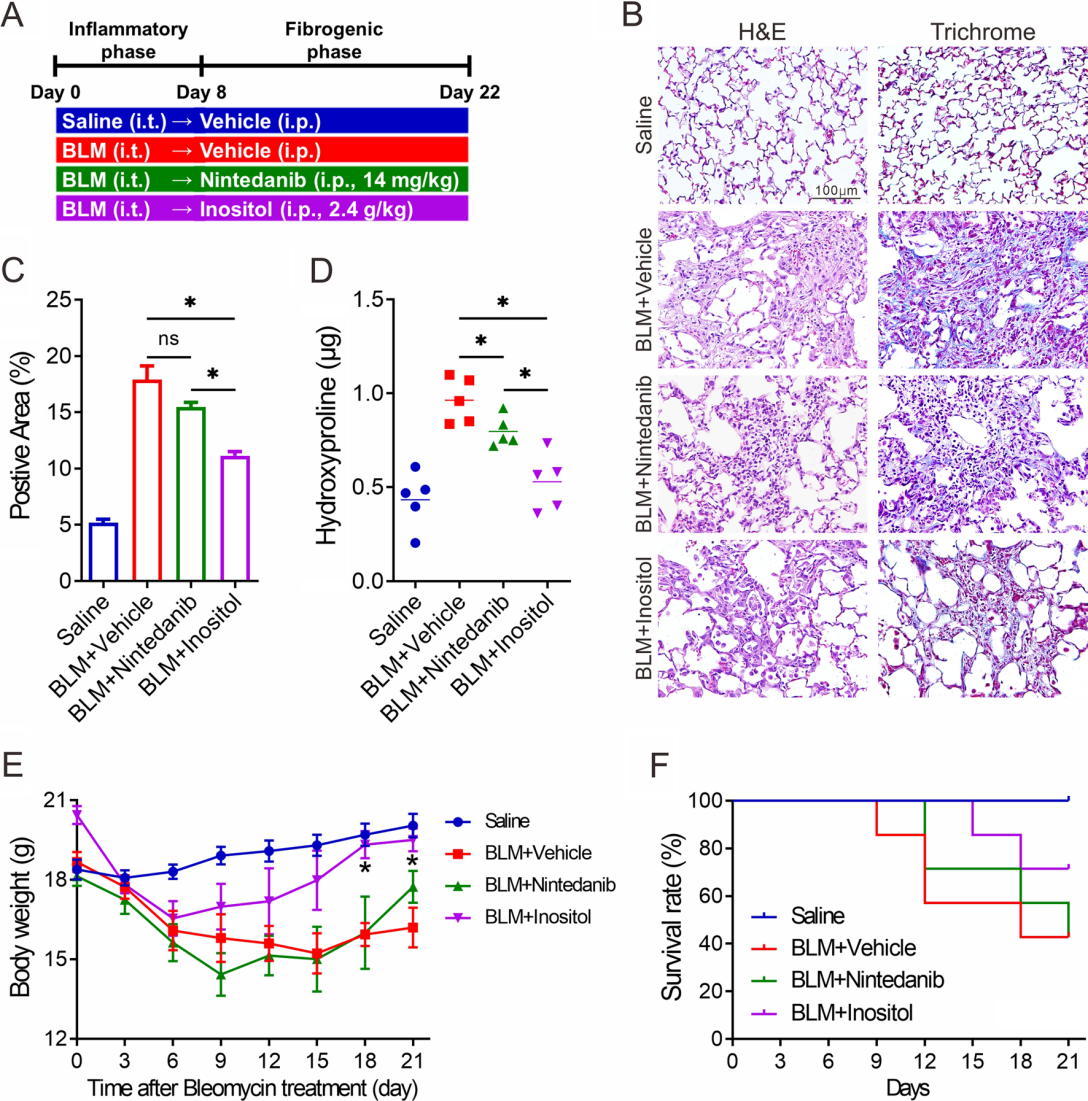
The anti-fibrotic effects of inositol on bleomycin-induced pulmonary fibrosis. **A** Schematic diagram of the sequence of events in bleomycin-induced pulmonary fibrosis. C57BL/6J mice (n = 7 per group) received intratracheal injection of saline or bleomycin (BLM) followed by vehicle, nintedanib, or inositol treatments. **B** Representative hematoxylin and eosin (H&E) and Masson’s trichrome staining of lung tissue from vehicle-, nintedanib- and inositol-treated mice with a single dose of bleomycin challenge. **C** Semiquantitative fibrosis evaluation of positive staining on histological Masson’s trichrome-stained sections of mouse lung. Fibrosis score is presented as the percentage of the positive staining area per high-powered field. Quantitative analysis of 6–12 high-powered fields per lung was performed by using ImageJ software (mean ± SE, n = 5, *p < 0.05) ns: non-significant. **D** Hydroxyproline content in the right lung homogenate samples detected by the hydroxyproline ELISA assay. Data are presented as mean with individual values (mean ± SE, n = 5, *p < 0.05). **E** The body weights of mice were measured at indicated days (mean ± SE, n = 5, *p < 0.05 versus BLM + vehicle group). **F** Kaplan-Meier analysis of the overall survival rates of vehicle-, nintedanib- and inositol-treated mice with a single dose of bleomycin challenge (*p* = 0.08, log-rank test; n = 7)

Given the role of inositol in suppressing fibrogenic activity, we next tested the feasibility of inositol supplementation as an antifibrotic therapy in the bleomycin-induced pulmonary fibrosis mouse model. To ascertain the therapeutic effect of inositol on pulmonary fibrosis, inositol supplementation was administered during the early fibrotic phase of the model. 8 days after an intratracheal instillation of bleomycin, mice were administered with either vehicle (PBS), nintedanib, or inositol intraperitoneally (i.p.) every two days. In total, there were four groups: (1) saline; (2) bleomycin plus vehicle; (3) bleomycin plus nintedanib (14 mg/kg); and (4) bleomycin plus inositol (2.4 g/kg). Figure 6A summarizes the experimental procedure and timelines for inositol supplementation in this study. After 14 days of drug treatment, lungs were collected and processed for histological and biochemical analysis. Bleomycin-exposed mice showed extensive structural changes in the lungs, whereas significant decreases of fibroblastic lesions and deposited extracellular matrix were seen in the lungs from mice with bleomycin exposure and inositol treatment (Fig. 6B, C). Likewise, bleomycin-induced upregulation of hydroxyproline levels was significantly suppressed in the bleomycin plus inositol group (Fig. 6D). We also observed a loss of body weight in the mice exposed to bleomycin plus vehicle, but not in the bleomycin-exposed mice with i.p. inositol supplementation (Fig. 6E). Compared to the bleomycin plus nintedanib group, i.p. inositol supplementation appeared to improve body weight of mice after bleomycin exposure. Surprisingly, only inositol-treated mice exhibited a higher percentage survival after bleomycin challenge, while nintedanib-treated mice did not (Fig. 6F). Altogether, these results suggest a potential therapeutic application of inositol supplementation in the treatment of pulmonary fibrosis.

## Discussion

Targeting altered fibrometabolism is an emerging therapeutic strategy for halting pulmonary fibrosis. The metabolic reprogramming that accompanies the development of fibrogenesis creates targetable differences between IPF (fibrotic or activated) lung fibroblasts and normal fibroblasts, which may be exploited for therapy [13–16]. Our prior studies revealed dysregulated expression of argininosuccinate synthase 1 (ASS1), a rate-limiting enzyme in the urea cycle, in IPF lung fibroblasts. Although we previously developed an arginine starvation strategy and demonstrated the therapeutic potential of arginine deiminase (ADI) in pulmonary fibrosis [16], many challenges remain to be resolved, especially the problem of induced ADI resistance [17, 18]. To develop a novel strategy to target arginine-dependent pulmonary fibrosis, we have conducted metabolomics studies and characterized the metabolic landscape of fibrotic lung fibroblasts mediated by ASS1. This current work identifies inositol as an antifibrotic metabolite in lung fibroblasts. In addition, we present a proof of concept for the use of inositol to suppress inositol-driven signaling and subsequent fibrotic activities in IPF lung fibroblasts as well as in bleomycin-induced pulmonary fibrosis in mice.

Our metabolomics studies demonstrated that a number of amino acid metabolic pathways (e.g., arginine and proline metabolism, inositol phosphate metabolism) were dysregulated in IPF and/or ASS1-deficient lung fibroblasts. Given that *de novo* arginine biosynthesis is suppressed in fibrotic lung fibroblast cells due to ASS1 deficiency [16], fibrotic lung fibroblasts rely on extracellular arginine to continuously supply the urea cycle and produce ornithine. Since ornithine has been revealed to positively regulate glucose-6-phosphatase (G6Pase) transcription [36], we believe that higher ornithine levels in fibrotic lung fibroblasts increase G6Pase expression, which in turn promotes hydrolysis of glucose 6-phosphate (G6P) and production of glucose. Our metabolomics data showing that elevated ornithine was concomitant with decreased G6P and increased glucose in IPF lung fibroblasts with ASS1 loss (Fig. 1D) further supports this notion. As noted above, given a crucial role of the G6P pathway for inositol biosynthesis, it is reasonable to expect that ASS1 deficiency contributes to dysregulation of inositol metabolism in IPF fibroblasts through its influence on the G6P pathway.

In general, ornithine breakdown feeds into the putrescine or proline pathway. A previous metabolomic study on IPF and normal lung tissues showed an increase in both putrescine and 4-hydroxyproline levels in lung tissues from IPF patients [11]. In contrast to IPF lung tissues, we found that IPF lung fibroblasts exhibited a reduction of putrescine and an elevation of 4-hydroxyproline, suggesting that ornithine metabolism favors proline synthesis in fibrotic lung fibroblasts. We also discovered dysregulation of the inositol-related metabolic pathways (inositol phosphate metabolism and phosphatidylinositol signaling system) and a shift in metabolite abundance from myo-inositol to inositol-4-monophosphate in ASS1-deficient cells, suggesting dysregulated inositol metabolism in fibrotic lung fibroblasts. These data support the notion of the ASS1-inositol axis in fibrometabolism, where inositol-associated metabolic pathways are rewired in response to ASS1 loss and modulate lung fibroblast activation and pulmonary fibrosis.

While we did observe a general downregulation of glycolytic intermediates in IPF fibroblasts, several studies on metabolomics of human lung tissues have demonstrated augmentation of glycolysis in IPF lung tissues compared to normal tissues [37, 38]. Lactic acid, also known as lactate, in particular was found to be elevated in IPF lungs [11, 37, 38]. Kottmann et al’s investigation of the mechanism points to the effect of increased lactic acid levels in lung tissues on TGF-β activation, myofibroblast differentiation, and pulmonary fibrosis [37]. The findings from the Liu group also support the notion of an increase in glycolysis in IPF lung tissues and upregulation of key glycolytic enzymes in lung myofibroblasts [38]. In Bernard et al’s study, an induction of mitochondrial biogenesis and aerobic glycolysis was demonstrated to occur in TGF-β-induced myofibroblast differentiation [39]. In contrast, our data showed a decrease of lactic acid in IPF lung fibroblasts. This is likely due to intrinsic differences between tissues and cells. In our study, we analyzed the metabolic profiling of lung fibroblasts rather than that of whole lung tissues. Lung fibroblasts derived from IPF patients are heterogeneous and consist of several subpopulations of fibroblasts including proliferative/aggressive fibroblasts and myofibroblasts [33–35]. Since most IPF lung fibroblasts used in this work lack ASS1 expression and the aforementioned studies mainly focused on myofibroblast differentiation [37–39], it would not be illogical that our observations in the metabolomics data differ from prior observations by other groups. Our findings of reduced lactic acid in IPF lung fibroblasts suggest that the products of glycolysis in ASS1-deficient lung fibroblasts may be shuttled towards other pathways, instead of lactic acid production. Despite a general decline of glycolysis in IPF fibroblasts, an increase in sorbitol and fructose may be alternative carbon sources for these fibrotic cells.

As dysregulated inositol was noted in IPF lung fibroblasts, most of which are deficient in ASS1, inositol-derived metabolites and critical components of inositol-mediated pathways were likely altered. As expected, we found that IPF fibroblasts with ASS1 deficiency display downregulated inositol level and upregulated expression of several enzymes (i.e., MIOX and CDIPT) involved in inositol catabolism and phosphatidylinositol metabolism, suggesting that ASS1-deficient lung fibroblasts tend to consume and/or eliminate most of cellular inositol. Given that inositol catabolism and phosphatidylinositol metabolism participate in generation of second messengers such as PIP2, IP3, and DAG as well as activate the downstream signaling cascades, it was not surprising to observe the activation of EGFR and PKC-mediated signaling molecules in ASS1-deficient cells due to an increase in inositol breakdown and elimination. Of these signaling molecules, EGFR, AKT, and STAT3 have been reported to participate in TGF-β-induced myofibroblast differentiation [40–42] and are being considered as potential therapeutic targets for IPF [27, 42–44]. Furthermore, our prior research revealed that targeting MARCKS activity in IPF lung fibroblasts with elevated α-SMA expression led to a reduction in COL1A1 and FN1 expression, AKT activity, and PIP3 levels [26]. Therefore, we propose that MARCKS, a major substrate of cPKCs, might potentiate COL1A1 and α-SMA expression in IPF fibroblasts. Based on these findings, it is plausible that the pathway underlying the facilitation of COL1A1 and α-SMA expression in ASS1 deficiency (with lower inositol) involves the activation of EGFR, AKT, STAT3, and MARCKS (cPKCs) pathways. However, further research is needed to explore the specific association between these pathways and COL1A1 and α-SMA expression in the context of inositol reduction.

Our current study has confirmed that ASS1 deficiency acted in parallel with decreased cellular inositol in IPF lung fibroblasts, showing the antifibrotic potential of cellular inositol abundance in cells. In cell-based studies, we found downregulation of inositol-related signalosomes (e.g., EGFR and PKC signaling) when “extracellular” inositol was added to increase “intracellular” levels of inositol in IPF fibroblasts and/or ASS1-knockdown normal fibroblasts. As mentioned earlier, fibrotic lung fibroblasts with ASS1 loss favor the state of lower cellular inositol, and there is a theoretical possibility that ASS1-deficient fibroblasts decrease inositol-associated signaling activity in order to reduce and/or avoid additional inositol uptake from outside the cell and sustain cell survival in response to high levels of extracellular inositol. Although downregulation of inositol-associated signaling pathways may act as a protective mechanism to overcome excess inositol entry into cells, we cannot completely rule out the possibility of the pathway crosstalk effect by inositol supplementation. In addition to binding to inositol transports, “extracellular” inositol may interact with receptors and/or kinases which in part regulate the inositol-associated signaling molecules.

Consistent with our observations, a handful of reports have confirmed that inositol supplementation suppresses the PI3K/AKT pathway in cancer cells [45–48]. Inositol plays a crucial role as a precursor of second messengers and regulates Ca^2+^ signaling through the PIP2 to IP3 circuit. An increase in calcium levels has been reported to activate calcium-dependent proteases such as calpains, which can induce apoptosis by activating caspase-3 in cancer cells [49, 50]. Inositol hexaphosphate (IP6), a phosphate metabolite of inositol, has demonstrated anti-cancer properties by inducing apoptosis in various cancers [46, 51]. However, the role of inositol in fibroblast apoptosis remains unknown and requires further investigation. Moreover, inositol has been documented to hinder epithelial-mesenchymal transition (EMT), a known fibrotic process, through upregulation of E-cadherin and downregulation of metalloproteinase-9 and snail family transcriptional repressor 1 levels [45]. Most significantly, we have demonstrated that inositol not only inhibited fibrotic molecules induced by ASS1 loss (e.g., COL1A1 and α-SMA) but also repressed cell invasiveness in IPF fibroblasts. In addition, our bleomycin-exposed mice treated with inositol had a significant improvement on histologic fibrosis and collagen deposition. Impressively, inositol supplementation supported body weight recovery in diseased mice with lung fibrosis. Weight loss is a critical issue in IPF patients as approximately 20% of IPF patients experience more than 5% unintended weight loss, and this weight loss is associated with worse IPF prognosis over time [52]. Despite mild efficacy in relieving the disease, adverse effects are common with the approved IPF therapies (nintedanib and pirfenidone) and both drugs are limited in halting lung fibrosis or in improving overall mortality [7, 8, 53]. In this work, inositol supplementation shows an inhibitory effect on lung fibrosis, weight loss, and overall mortality in bleomycin-exposed mice, indicating the possibility of inositol supplementation as a viable antifibrotic therapeutic strategy for IPF.

Inositol has been evaluated in both pre-clinical and clinical studies for various diseases, and many toxicity studies suggest that taking lower dosages of inositol are relatively safe [54]. Given the extensive safety data in clinical trials and our findings on preclinical tests, inositol presents itself as a promising antifibrotic agent, all of which will facilitate quicker translation to clinical trials and potential regulatory approval. In addition to its antifibrotic activity, the effect of inositol in mitigating lung fibrosis of bleomycin-exposed may be mediated partly through anti-inflammatory effects. In the following phase II trial of smokers with bronchial dysplasia, 6-month inositol intervention was reported to reduce IL-6 level in bronchoalveolar lavage fluid of treated subjects, and the treatment significantly decreased PI3K activation among those with complete response [23]. A decrease in IL-6 promotes macrophage polarization and subsequently lowers STAT3 activity, thereby reducing tumor development in cancer-prone mice fed with inositol diet [55]. Identifying whether inositol modulates the cytokine profile and immune cell populations during fibrogenesis is a promising area of future study. However, work in understanding how inositol influences the immune landscape within the fibrotic microenvironment is beyond the scope of this study but merits further investigation.

## Conclusions

To our best knowledge, our study is the first report of the functionality of inositol in fibroblast cells and the novel ASS1-inositol-signaling circuit. This study presents a novel function of inositol as a therapeutic treatment for lung fibrosis. Metabolomic data provide evidence of crosstalk between biosynthesis (ASS1) and inositol metabolic pathways in IPF lung fibroblasts. Our cell-based studies reveal a novel antifibrotic metabolite in fibrotic lung fibroblasts. In conjunction with the established clinical profiles of inositol, our preclinical findings support the translational potential of inositol in future IPF clinical studies. In summary, targeting inositol-related metabolic and signaling pathways driven by ASS1 deficiency represent an effective approach to halt lung fibrosis, and our study offers evidence of inositol supplementation as a viable and potential therapeutic avenue for IPF.

## Supplementary Information

**Additional file 1**: **Additional methods**. **Table S1**. The list of primer sequences for target genes utilized in quantitative real-time PCR analysis. **Figure S1**. Related to Fig. 1. **Figure S2**. Related to Fig. 1. **Figure S3**. Related to Fig. 2. **Figure S4**. Related to Fig. 2.

## Data Availability

For all data requests, please contact the corresponding authors.

## Abbreviations

GLC: Glucose
G6P: Glucose 6-phosphate
G3P: Glyceraldehyde 3-phosphate
3PG: 3-phosphoglycerate
2,3-BPG: 2,3-Bisphosphoglyceric acid
PEP: Phosphoenolpyruvate
PYR: Pyruvate
FRU: Fructose
F1P: Fructose 1-phosphate
Ru5P: Ribulose-5-phosphate
MAN: Mannose
SER: Serine
LYS: Lysine
α-AAA: α-aminoadipic acid
CIT: Citrate
α-KG: α-ketoglutarate
GLN: Glutamine
2-HG: 2-hydroxyglutarate
SSA: Succinic semialdehyde
FUM: Fumarate
ASP: Aspartate
ASN: Asparagine
ORN: Ornithine
4HyP: 4-hydroxyproline
PUT: Putrescine
SPD: Spermidine
SPR: Spermine
INPP4A: Inositol polyphosphate-4-phosphatase type I A
IMPA2: Inositol monophosphatase 2
ISYNA1: Inositol-3-phosphate synthase 1
MIOX: Myo-inositol oxygenase
CDIPT: CDP-diacylglycerol-inositol 3-phosphatidyltransferase
