# Supplementary material for: Inositol possesses antifibrotic activity and mitigates pulmonary fibrosis
Source: Respir Res. 2023 May 16;24:132. doi: 10.1186/s12931-023-02421-6 (PMC10189934; doi:10.1186/s12931-023-02421-6)
Supplement: Supplementary file 1 — Additional file 1: Additional methods. Table S1. The list of primer sequences for target genes utilized in quantitative real-time PCR analysis. Figure S1. Related to Fig. 1. Figure S2. Related to Fig. 1. Figure S3. Related to Fig. 2. Figure S4. Related to Fig. 2. [file 12931_2023_2421_MOESM1_ESM.docx]

Additional file 1

**Inositol possesses antifibrotic activity and mitigates pulmonary fibrosis**

Ji-Min Li, Wen-Hsin Chang, Linhui Li, David C. Yang, Ssu-Wei Hsu,

Nicholas J. Kenyon and Ching-Hsien Chen*

**Methods**

**Reagents and antibodies**

Dulbecco’s modified Eagle’s medium (DMEM), fetal bovine serum (FBS) and penicillin-streptomycin were purchased from Gibco, Thermo Fisher Scientific (Waltham, MA, USA). Myo-inositol was purchased from Alfa Aesar (Tewksbury, MA, USA). Bleomycin was purchased from EMD Millipore (Billerica, MA, USA). Anti-phospho-EGFR (Tyr1068), anti-EGFR, anti-phospho-AKT (Ser473), anti-AKT, anti-phospho-STAT3 (Tyr705), anti-STAT3, anti-COL1A1 and anti-GAPDH were purchased from Cell Signaling Technology (Danvers, MA, USA). Anti-ASS1 and RNAiMAX Transfection Reagent were purchased from Invitrogen, Thermo Fisher Scientiﬁc (Waltham, MA, USA). Anti-phospho-MARCKS (Ser158), anti-MARCKS and Cell Counting Kit 8 (WST-8/CCK8) were purchased from Abcam (Cambridge, MA, USA). Anti-smooth muscle actin (α-SMA) was purchased from ARP (American Research Products, Inc., Waltham, MA, USA). Recombinant Human TGF-β was purchased from BioLegend (San Diego, CA, USA). Myo-Inositol Assay Kits were purchased from Abcam (Cambridge, MA, USA). Hydroxyproline Assay Kits were purchased from MilliporeSigma (Burlington, MA, USA).

**Cell culture and transfection**

Human IPF lung fibroblast cells were derived from patients who underwent biopsies and histologically confirmed IPF at the UC Davis Medical Center (Sacramento, CA, USA) as previously described [1]. The diagnoses of all patients with IPF were validated by disease history, physical examination, lung function tests, microscopic analysis of lung tissue, and the characteristic of usual interstitial pneumonia (UIP) on typical high-resolution chest computed tomography. The protocol for human tissue investigation was approved and reviewed by the Institutional Review Board of the UC Davis Health System. Human normal lung ﬁbroblasts were established from histologically normal lung tissues adjacent to tumor or human airway samples of individual postmortem. The IPF fibroblast cell line LL-97A was purchased from the American Type Culture Collection (ATCC) (Manassas, VA, USA). All lung fibroblast cells were cultured in high-glucose DMEM supplemented with 10% fetal bovine serum (FBS) and 1% penicillin-streptomycin at 37°C with a humidified atmosphere of 5% CO_2_. For siRNA transfection, ON-TARGETplus control siRNA and ON-TARGETplus ASS1 siRNAs containing 4 independent ASS1-specific siRNAs were designed and synthesized by Dharmacon, Horizon Discovery (Lafayette, CO, USA). Cells were grown to optimal confluence and transfected with 60 nM of control siRNA or ASS1 siRNA by using RNAiMAX Transfection Reagent, according to the manufacturer’s instructions (Invitrogen). After siRNA transfection in Opti-MEM for 8 hours, cells were incubated in fresh complete medium for at least 48 hours.

**Untargeted GC-TOF MS analysis**

Frozen lung fibroblast samples were submitted to West Coast Metabolomics Center at UC Davis, and the cells were analyzed using gas chromatography/time-of-flight mass spectrometry (GC-TOFMS) analysis as previously described [2]. In brief, 0.5 μL of sample was injected with 25 splitless time into an Angilent 6890 GC (Angilent Technologies, Santa Clara, CA, USA) equipped with a 30 m length × 0.25 mm Restek corporation Rtx-5Sil MS column with 0.25 µm film thickness and 1 mL/min Helium gas flow. The oven temperature was programmed as follows: 50°C (1 min), 20°C/min to 330°C, held constant for 5 min. Mass spectrometry data were acquired using a Leco Pegasus IV mass spectrometer (Leco Corporation, St. Joseph, MI, USA) with unit mass resolution at 17 spectra s -1 from 80-500 Da at -70 eV ionization energy and 1800 V detector voltage with a 230°C transfer line and a 250°C ion source. Raw data were preprocessed by ChromaTOF version 2.32 for baseline subtraction, deconvolution, and peak detection. Binbase was employed for metabolite annotation and reporting. For data analysis, partial least squares discriminant analysis (PLS-DA) and variable importance in projection (VIP scoring) was calculated using all the metabolites from the same dataset, and no missing value replacement was performed. PLS-DA figures were generated using MetaboAnalyst 5.0 (http://www.metaboanalyst.ca/). MetaboAnalyst 5.0 was also applied to perform enrichment analysis. Metabolites from the GC-MS dataset were imported into the quantitative pathway analysis module. For quantitative enrichment analysis, MetaboAnalyst 5.0 measured both the counts (presence/absence) as well as Q-statistics to identify biologically meaningful patterns. Enrichment pathway figures were generated using MetaboAnalyst 5.0.

**Myo-inositol assays**

The content of inositol in lung fibroblast cells was measured by using myo-inositol assays according to the manufacturer’s protocol (Abcam, Cambridge, MA). Briefly, 5 × 10^5^ cells were collected and homogenized in 100 μL of inositol assay buffer. After the cell homogenates were centrifuged at 10,000 × *g* for 5 min at 4°C, the supernatant was transferred into a 1.5-mL tube. 2 µL of sample clean-up mix was added and incubated for 1 hour at 37°C. The samples were then transferred into the 10 kDa spin column and centrifuged 10,000 × *g* for 20 min at 4°C. 20 µL of filtrate was added into 96-well white plate and mixed with 50 µL of reaction mix. After samples were incubated for 30 min at 37°C, the fluorescence was measured at excitation (535 nm) and emission (587 nm) by using an ELISA reader (Synergy H1; BioTek Instruments [BioTek], Winooski, VT, USA). The inositol concentration was extrapolated from the inositol standard curve according to the manufacturer’s instructions.

**Immunoblotting**

The ﬁbroblast cells were collected and prepared as the whole-cell lysates with lysis buffer (50 mM Tris-HCl [pH 7.4], 1% Triton X-100, 10% glycerol, 150 mM NaCl, 1 mM EDTA, 20 μg/mL leupeptin, 1 mM PMSF, and 20 μg/mL aprotinin). Total proteins were separated via SDS-PAGE and transferred to polyvinylidene fluoride (PVDF) membranes. Immunoblotting was performed by using appropriate antibodies probed on the membranes, followed by visualization through an enhanced chemiluminescence method. The protein expression levels were quantified by ImageJ software (National Institutes of Health, Bethesda, MD, USA).

**Quantitative real-time PCR**

Total mRNA of fibroblast cells was extracted using the TRIzol reagent (Invitrogen, Carlsbad, CA, USA). cDNAs were synthesized from total mRNA using SuperScript III Reverse Transcriptase (Invitrogen). TATA-binding protein (TBP), a housekeeping gene, was used as the reference gene in quantitative real-time RT-PCR assay. The primers used were listed in Table S1. Quantitative real-time RT-PCR was analyzed using the SYBR Green system and performed according to manufacturer’s instructions of the ViiA 7 Real-Time PCR System (Applied Biosystems, Thermo Fisher Scientiﬁc, Waltham, MA, USA). The relative expression level of the target gene compared with that of TBP was defined as –ΔCT = –[CT*_target_* – CT*_tbp_*]. The target/TBP mRNA ratio was calculated as 2 ^–ΔCT^ × *K*, where *K* is a constant.

**Cell viability and colony formation assays**

Cells were trypsinized and seeded onto 96-well plates at a density of 3 × 10^3^ cells per well. The cell viability was determined using a Cell Counting Kit 8 (CCK8) assay (Abcam, Cambridge, MA, USA). After the indicated treatment of myo-inositol (0-40 mM) for 72 hours, 10 μL of the WST-8 Solution was added into each well, incubated for 4 h at 37°C. The absorbance was measured by using an ELISA reader (Synergy H1; BioTek Instruments [BioTek], Winooski, VT, USA) at 460 nm. For colony-forming assays, 500 cells were seed into 12-well plates, and maintained in fresh medium containing myo-inositol (0, 5, and 10 mM) for 14 days. The colonies were stained with 0.001% crystal violet, and stained colonies more than 0.5 mm in diameter were counted using an inverted microscope.

**Cell invasion assays**

Cell invasion assay was performed *in vitro* using Transwell chambers (8 mm pore size; Costar, Cambridge, MA, USA) as previously described [1, 3]. In brief, cells were pre-treated with 10 mM of myo-inositol for 48 hours, and 5 × 10^4^ cells were seeded into the upper chamber with the polyethylene terephthalate (PET) ﬁlters coated with Matrigel (Becton Dickinson, Franklin Lakes, NJ, USA). 0.5 mL of growth medium containing 10 mM of myo-inositol was added to both the upper and lower wells, and then incubated for 20 h at 37°C. Filters were swabbed with a cotton swab, ﬁxed with methanol, followed by stained with Giemsa solution (Millipore Sigma, Burlington, MA, USA). The cells attached to the underside of the ﬁlter were counted under a light microscope (10× magniﬁcation).

**Bleomycin-induced pulmonary fibrosis model**

8-week-old C57BL/6J female mice purchased from The Jackson Laboratory (Bar Harbor, ME, USA) were housed 4 mice per cage and fed with rodent laboratory chow *ad libitum*. Saline or bleomycin was intratracheally administered to C57BL/6J mice as previously described [1, 3, 4]. Briefly, mice were anesthetized with 5% isoﬂurane and intratracheally instilled with sterile saline or 0.005 U/g bleomycin (EMD Millipore, Billerica, MA, USA) on day 0. From day 8 in the early fibrogenic phase, bleomycin-challenged mice received intraperitoneal injections every two days with vehicle, nintedanib (14 mg/kg), or myo-inositol (or inositol, 2.4 g/kg). 22 days after bleomycin insult, these mice were euthanized, and lungs were harvested for histological and collagen content analysis. The procedures of all mouse experiments were approved by the Institutional Animal Care and Use Committee (IACUC) of UC Davis.

**Hydroxyproline assays**

The amount of collagen in mouse lung tissues was measured using the hydroxyproline assay kit according to the manufacturer’s protocol (MilliporeSigma). In brief, 10 mg of lung tissue in 100 μL water was homogenized using the sonicator (VC50 Vibra-Cell; Sonics and Materials, Newtown, CT, USA) and transferred into the glass Wheaton ampules (Wheaton, Millville, NJ, USA). 100 μL of hydrochloric acid (12M) was added and hydrolyzed for 3 h at 120°C. Each sample was mixed and centrifuged at 10,000 × *g* for 3 min, and then transferred 10-50 μL of the supernatant to the 96-well flat-bottomed plate. After samples were all dried out in a 60°C oven, 100 μL of Chloramine T/Oxidation buffer mixture was added and incubated for 5 min at room temperature. Thereafter, 100 μL of the diluted 4-(dimethylamino) benzaldehyde (DMAB) reagent was added and incubated for 90 min at 60°C. The absorbance was measured at 560 nm, and the concentration of hydroxyproline for each sample was determined from the hydroxyproline standards according to the manufacturer’s instructions (MilliporeSigma).

**Table S1. The list of primer sequences for target genes utilized in quantitative real-time PCR analysis.**

| **Target Gene** | **Primer Sequence** |
| --- | --- |
| **INPP4A** | F: 5’-CTTGAGAGGACACTCGCCATCT-3’  R: 5’-CTTGGAGGCAATGTAGTCAGGC-3’ |
| **IMPA2** | F: 5’-CGGATGCCTATTACCAGTTTGGC-3’  R: 5’-CAACCACTCTGCAAGCCATGAG-3’ |
| **ISYNA1** | F: 5’-GCCAGACCAAAGTCAAGTCCGT-3’  R: 5’-CTTAGAGCGGAACTGCAATGGC-3’ |
| **CDIPT** | F: 5’-GCTCTGTTCACCTTGTGTGCTG-3’  R: 5’-TGATGAGCGACTTCAGCAAGGC-3’ |
| **MIOX** | F: 5’-CACCAGACAGTGGACTTCGTCA-3’  R: 5’-GGTCCGACTCATCCACCAGCC-3’ |
| **SLC2A13** | F: 5’-GTCTGGCTTGTTGAGAAGGTGG-3’  R: 5’-CGTTCTGACCTGACGGAGCTAT-3’ |
| **SLC5A3** | F: 5’-GCCAGTACCATATTCACCCTCG-3’  R: 5’-CATCTCCACGATGATTGGCACC-3’ |
| **SLC5A11** | F: 5’-CCAAACTCGTGCTGGAACTCCT-3’  R: 5’-GTGAAGATGGTGCTGGCACTGT-3’ |
| **α-SMA** | F: 5’-TCCTCATCCTCCCTTGAGAA-3’  R: 5’-ATGAAGGATGGCTGGAACAG-3’ |
| **TBP** | F: 5’-CACGAACCACGGCACTGATT-3’  R: 5’-TTTTCTTGCTGCCAGTCTGGAC-3’ |


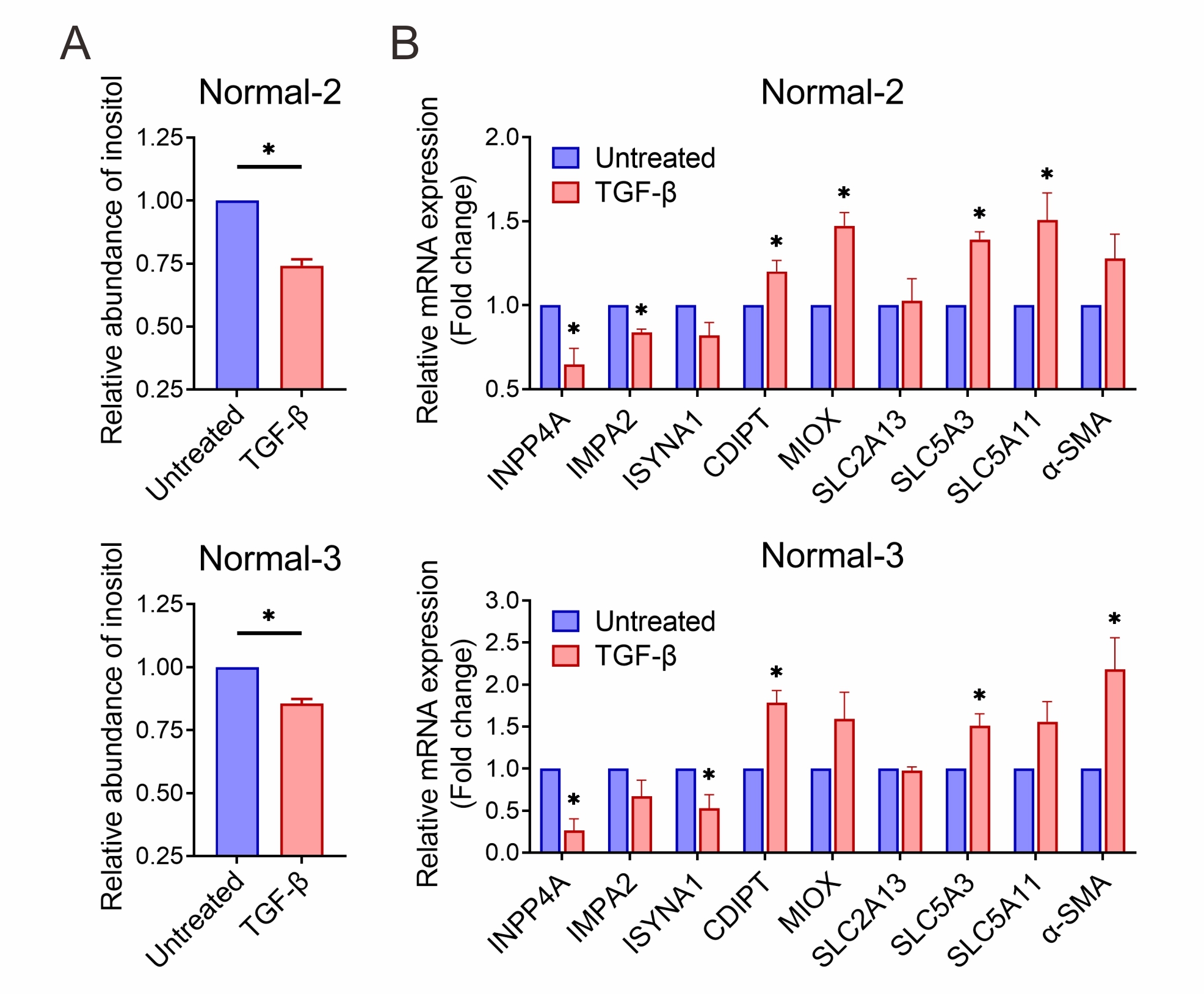


**Figure S1. Related to Figure 1**

(**A**) Normal fibroblasts (Top: Normal-2; Bottom: Normal-3) were treated with 10 ng/mL of TGF-β for 72 hours and subjected to an inositol assay (mean ± SE, n=3, **p* < 0.05 versus untreated group). (**B**) The mRNA expression levels of the enzyme associated with inositol biosynthesis (INPP4A, IMPA2, and ISYNA1), inositol catabolism and phosphatidylinositol metabolism (CDIPT and MIOX) as well as the gene expression of the inositol transporters (SLC2A13, SLC5A3, and SLC5A11) in normal fibroblasts (Top: Normal-2; Bottom: Normal-3) treated with or without TGF-β. The myofibroblast marker α-SMA was also determined. Data are expressed as mean ± SE (n=3, **p* < 0.05 versus untreated group).


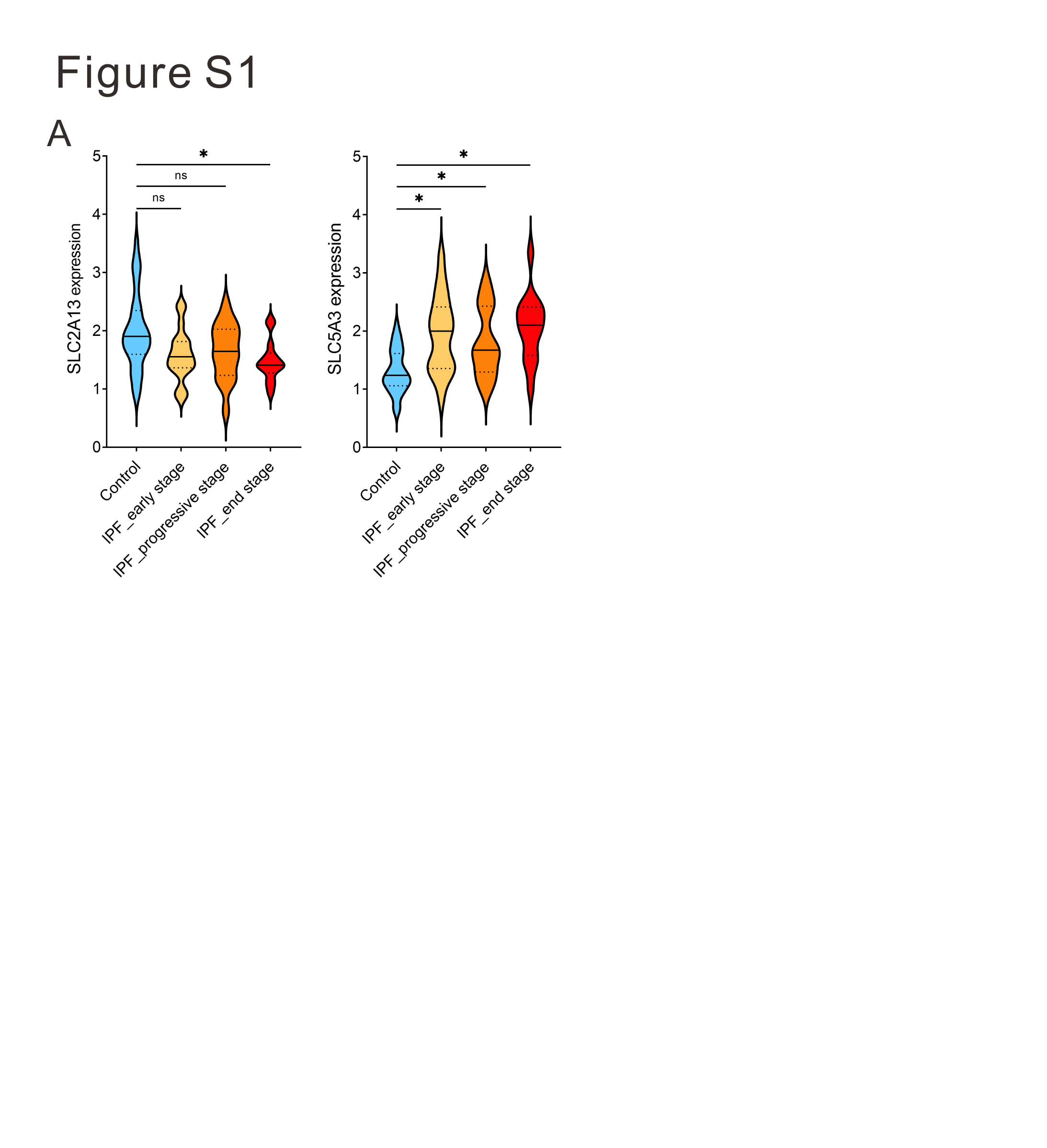


**Figure S2. Related to Figure 1**

(**A**) Myo-inositol (or inositol) transporter gene expression in different stages of IPF. The expression level of SLC2A13 and SLC5A3 genes in control (blue) and different disease stages of IPF, including early (yellow), progressive (orange), and end (red) stages, from the GSE124685. *, *p*-value < 0.05.


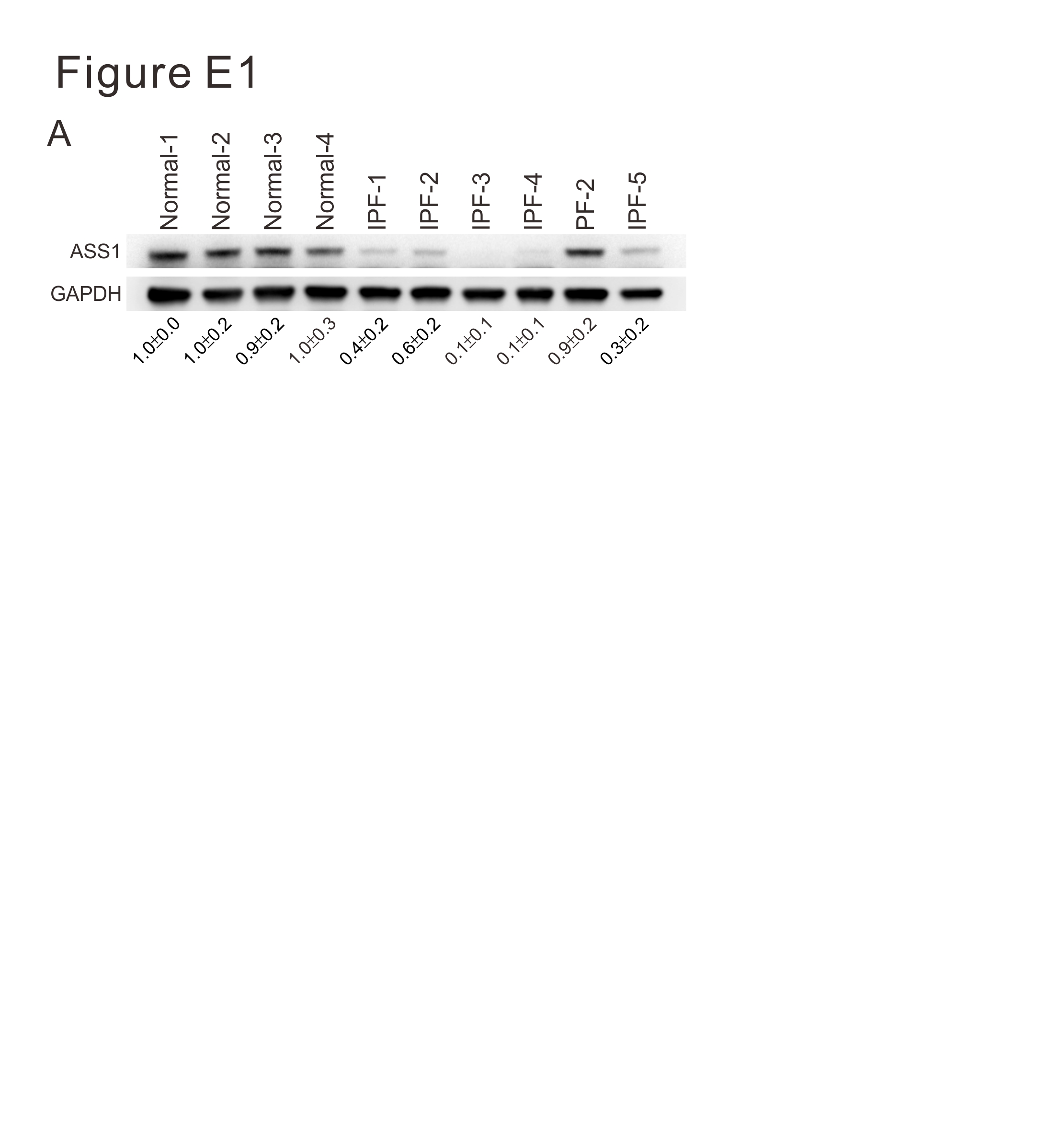


**Figure S3. Related to Figure 2**

(**A**) Expression levels of ASS1 in four primary normal fibroblasts (Normal-1, -2, -3, and -4), five IPF fibroblast cells (IPF-1, -2, -3, -4, and -5) and one interstitial lung disease fibroblast cell line (PF-2) as stained with an anti-ASS1 antibody.


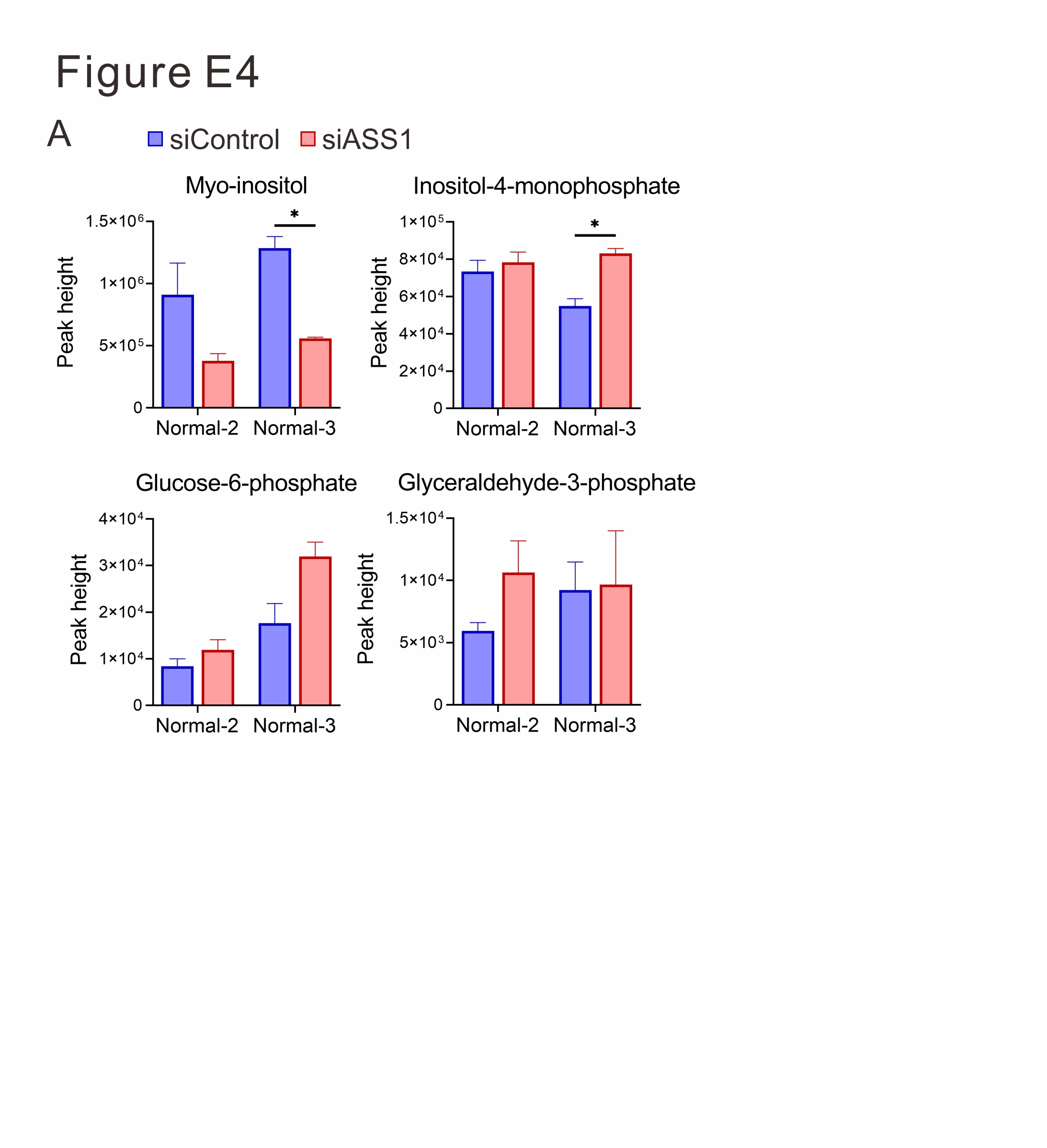


**Figure S4. Related to Figure 2**

(**A**) The relative levels of dysregulated metabolites (inositol, inositol-4-monophosphate, glucose-6-phosphate, and glyceraldehyde-3-phosphate) associated with inositol metabolism in normal fibroblasts receiving control or ASS1-specific siRNAs for 72 hours are presented as peak height after normalization to cell count (mean ± SE, n=3, **p* < 0.05 versus siControl).

**References**

1. Li JM, Yang DC, Oldham J, Linderholm A, Zhang J, Liu J, Kenyon NJ, Chen CH. Therapeutic targeting of argininosuccinate synthase 1 (ASS1)-deficient pulmonary fibrosis. Mol Ther. 2021;29:1487-1500.

2. Ding J, Ji J, Rabow Z, Shen T, Folz J, Brydges CR, Fan S, Lu X, Mehta S, Showalter MR, et al. A metabolome atlas of the aging mouse brain. Nat Commun. 2021;12:6021.

3. Yang DC, Li JM, Xu J, Oldham J, Phan SH, Last JA, Wu R, Chen CH. Tackling MARCKS-PIP3 circuit attenuates fibroblast activation and fibrosis progression. FASEB J. 2019;33:14354-14369.

4. Yang DC, Gu S, Li JM, Hsu SW, Chen SJ, Chang WH, Chen CH. Targeting the AXL Receptor in Combating Smoking-related Pulmonary Fibrosis. Am J Respir Cell Mol Biol. 2021;64:734-746.
